# Epidemiological and clinical characteristics of fall-related injuries: a retrospective study

**DOI:** 10.1186/s12889-020-09268-2

**Published:** 2020-07-29

**Authors:** Ahammed Mekkodathil, Ayman El-Menyar, Ahad Kanbar, Suhail Hakim, Khalid Ahmed, Tariq Siddiqui, Hassan Al-Thani

**Affiliations:** 1 Clinical Research, Trauma & Vascular Surgery Section, Hamad General Hospital , PO Box 3050, Doha, Qatar; 2 Clinical Medicine, Weill Cornell Medical College, Doha, Qatar; 3 Department of Surgery, Trauma Surgery Section, Hamad General Hospital (HGH), Doha, Qatar; 4 Department of Surgery, Trauma &Vascular Surgery Section, HGH, Doha, Qatar

**Keywords:** Fall, Height, Trauma, Injury, Qatar

## Abstract

**Background:**

Fall-related injuries are important public health problem worldwide. We aimed to describe the epidemiological and clinical characteristics of fall-related injuries in a level 1 trauma center.

**Method:**

A retrospective analysis of Qatar Trauma Registry data was conducted on patients admitted for fall-related injuries between 2010 and 2017. Comparative analyses of data by gender, age-groups and height of falls were performed to describe the epidemiological and clinical characteristics of patients, and in-hospital outcomes.

**Results:**

A total of 4040 patients with fall-related injuries were identified in the study duration which corresponds to the rate of 2.34 per 10,000 population. Although the rate of fall-related injuries decreased over the years, the average number of patients per year remained high accounting for 32% of the hospitalized patients with moderate to severe injuries. Most of the injuries affected the head (36%) followed by spines (29%) and chest (23%). Males were predominant (89%), more likely to fall at workplace, fall from a greater height and have polytrauma than females. The working age-group (20–59 years) constituted the majority of injured (73%) and were more likely to fall at workplace, and to fall from higher heights compared to the older adults who sustained more fall at home. Overall in-hospital mortality was 3%. Outcomes including longer hospital length of stay and mortality were generally correlated with the height of fall except for the fall at home.

**Conclusion:**

Fall-related injuries remain as significant burden even in a level 1 trauma center. Variations in the pattern of injuries by age, gender and height of fall provide important information for targeted preventive measures.

## Background

Injuries resulting from fall from height (FFH) are a growing public health problem worldwide. According to the World Health Organization (WHO), falls are the second leading cause of unintentional injuries resulting in deaths, with an estimated death rate of 646,000 individuals globally [1]. Each year, approximately 37.3 million falls occur globally which are usually severe and require medical attention. This leads to a loss of 17 million disability-adjusted life years (DALYs), i.e. loss of potential years of life due to premature death. Economic burden associated with fall injuries are substantial, especially among the older adults who may require hospitalization, long-term care and financial support [1]. Older adults over 70 years of age are at increased risk of fall-related mortality [1]. However, significantly higher mortality was reported among pediatric and very young adults aged 15–29 years who accounts almost half of the DALYs lost universally [1].

Epidemiology of fall-related injuries in Qatar remains understudied, however some hospital-based studies on moderate to severe injuries suggested that FFH was the key contributor for work-related injuries (nearly 50%), particularly among the 18–59 age group [2]. Incidence of fall-related injuries was estimated as 86.7 per 100,000 workers with a mortality rate of 8.44 per 100,000 workers costing over 4.4 million USD, with an average cost of 15,735 USD per patient which is in fact costs 15 times higher than that in the United States [1, 3]. Another study on injury burden among the pediatric population in Qatar revealed that fall at home was the most common mechanism among the age group of 0–4 years, leading to severe injuries requiring hospitalization [4]. Prevalence of fall in the older adults over 60 years based on primary healthcare visits in Qatar was estimated as 34% with recurrent falls in 53% of the subjects [5]. Albeit, there is scarcity of information on epidemiological and clinical characteristics of fall-related injuries in Qatar based on a nationally representative sample addressing all the age groups. Such studies will contribute to policy-making and developing preventive strategies.

Therefore, the primary objective of this study is to describe the epidemiology and trends of unintentional falls requiring hospitalization in Qatar. The study will also describe the clinical implications and in-hospital outcomes by gender, age group, and height of fall in the duration between 2010 and 2017 in a level 1 trauma center.

## Methods

### Setting and subjects

A retrospective study was conducted among patients admitted to the Hamad Trauma Center (HTC) of Hamad General Hospital (HGH) in Qatar. The study included all patients who had fall-related injuries (intentional and unintentional) in the duration between January 2010 and December 2017. The WHO definition of fall was followed in the current study, which refers fall as “inadvertently coming to rest on the ground or other lower level, excluding intentional change of position to lean on furniture, walls or other objects” [6]. Patients with mild injuries following fall and presented to the Emergency Department (ED), and discharged without admission were excluded from the analysis. Brought in Dead (BID) cases were also excluded from the study.

The HTC is the only tertiary care facility and level 1 trauma center in Qatar with 1500–1800 trauma admissions each year [7]. Data included in the study were nationally representative, retrieved from a prospectively maintained national trauma registry of trauma surgery section under the department of surgery in HGH. Qatar Trauma Registry is a mature database that participates in both National Trauma Data Bank and Trauma Quality Improvement Program of Committee on Trauma by the American College of Surgeons (TIQP-ACS). Ethical approval for the study was granted from the medical research center and institutional review board of Hamad Medical Corporation, Doha, Qatar (IRB#MRC-01-18-004).

### Data collection

Data on fall-related injuries required trauma admission in the study duration were collected from the Qatar Trauma Registry. The trauma registry prospectively records the fall data using codes by International Classification of Diseases-10th Revision (ICD-10) which classified unintentional falls into 20 subcategories (W00-W19).

Data collected included patients’ demographics such as age, gender, nationality and occupation; locations of falls including workplace, home or recreational-related; heights of fall in meters; body regions injured; vital signs; various injury scores and outcomes including length of stays in intensive care unit (ICU), ventilator and hospital length of stay (HLOS), and in-hospital mortality.

### Definitions

Consciousness following head injury was assessed using Glasgow Coma Scale (GCS) ranges from 3 to 15 in which GCS < 8 is severe, 9–12 is moderate and ≥ 13 is minor head injuries [8]. The Abbreviated Injury Scale (AIS) describe the severity of injuries at different body regions; the score ranges from 1 to 6, representing minor, moderate, serious, severe, critical and non-survivable injuries respectively from 1 to 6 [9]. AIS scores of 3 most severely injured body regions are squared and added together to estimate the Injury Severity Score (ISS) which provides an overall score for polytrauma [10]. The ISS score ranges from 0 to 75; 1–8 is major, 9–15 is moderate, 16–24 is serious, 50–74 is critical and 75 is non-survivable [10]. The Revised Trauma Score (RTS) provides information about patient triage based on the severity in terms of GCS, systolic blood pressure (SBP) and respiratory rate (RR), ranges from 0 to 12 in which 3–10 indicates immediate, 11 urgent and 12 delayed triages. RTS is calculated using following equation [11]:

$$ \mathrm{RTS}=0.9368\ \left(\mathrm{GCS}\right)+0.8326\ \left(\mathrm{SBP}\right)+0.2908\ \left(\mathrm{RR}\right) $$

Trauma and injury severity score (TRISS), is an index that determines probability of survival based on RTS, ISS and age of the patient; the higher value indicates better prognosis. The survival probability by TRISS is calculated using following formula [12].

Survival probability = 1/(1+ e^-b^) where b for blunt and penetrating injuries are

$$ {\displaystyle \begin{array}{l}{\mathrm{b}}_{\mathrm{Blunt}}=\hbox{-} 0.4499+0.8085\ \mathrm{x}\ \mathrm{RTS}\hbox{-} 0.0835\ \mathrm{x}\ \mathrm{ISS}\hbox{-} 1.7430\ \mathrm{x}\ \mathrm{Age}\ \mathrm{Index}\\ {}{\mathrm{b}}_{\mathrm{Penetrating}}=\hbox{-} 2.5355+0.9934\ \mathrm{x}\ \mathrm{RTS}\hbox{-} 0.0651\ \mathrm{x}\ \mathrm{ISS}\hbox{-} 1.1360\ \mathrm{x}\ \mathrm{Age}\ \mathrm{Index}\end{array}} $$

Population data of Qatar was collected from the official website of Ministry of Development Planning and Statistics, Qatar to estimate the rates of injuries in each year [13].

### Data analysis

Data were expressed as rates per 10,000 population, numbers, percentages, mean ± standard deviation or medians with interquartile range whenever appropriate. Percentage change in the rate of fall injuries per 10,000 population was calculated to express the pattern of burden of fall-related injuries in the study duration. Comparative analyses were performed by classifying patients into groups by gender; by different age-groups (0–19 years, 20–59 yrs. and ≥ 60 years); 20–45 vs 46–64 years and by height of fall in meters (< 1 m, 1.0–2.9 m,3.0–5.9 m and ≥ 6 m). Differences in categorical variables between groups were analyzed using Chi square tests or Fisher exact tests when observed cell values *n* < 5. The continuous variables between different gender groups were compared using student’s t tests and two-tailed *p* values < 0.05 were considered as significant. One-way ANOVA tests were performed for multiple comparisons of means between the groups using Bonferroni technique when equal variances were assumed with the mean difference is at significant level < 0.05. Data analysis was carried out using the Statistical Package for Social Sciences version 18 (SPSS Inc. Chicago, Illinois, USA).

## Results

There were 4040 patients of all age groups admitted to the HTC sustaining fall-related injuries over 8 years. Eighty-nine percent of the hospitalized patients were males. Nearly 77% of victims were transported to the hospital by ground emergency medical services (GEMS) whereas approximately 3% were transported by helicopter emergency medical services (HEMS).

The average number of fall-related admissions was 505 per year which represents 32% of the trauma admissions (505/1600). The average rate of fall-related injuries was estimated as 2.34 per 10,000 persons; males were affected at a higher rate (2.78 per 10,000 males) than females (1.00 per 10,000 females). A 39% decrease was estimated in the overall rate of fall-related injuries over the years from 2010 to 2017.

Table 1 shows the number and rate of hospitalized fall-related injuries in Qatar by gender across the years 2010–2017. A peak in the rate of fall-related injuries was seen in the year 2011 (overall rate 3.14 per 10,000 population), especially among the males (3.77 per 10,000 males) whereas a peak in fall injuries among females was observed in 2010 (1.43 per 10,000 females).

**Table 1:** Number and rate of hospitalized fall-related injuries in Qatar by gender across the years 2010–2017

Year	Number of fall-related injuries			Estimated population in Qatar			Rate of fall-related injuries per 10,000		
	Males	Females	Total	Males	Females	Total	Males	Females	Overall
**2010**	432	60	492	1,296,110	418,988	1,715,098	3.33	1.43	2.87
**2011**	486	58	544	1,288,590	444,127	1,732,717	3.77	1.31	3.14
**2012**	450	58	508	1,355,199	477,704	1,832,903	3.32	1.21	2.77
**2013**	436	53	489	1,477,632	526,068	2,003,700	2.95	1.01	2.44
**2014**	439	53	492	1,652,037	564,143	2,216,180	2.66	0.94	2.22
**2015**	487	50	537	1,840,643	597,147	2,437,790	2.65	0.84	2.20
**2016**	459	43	502	1,975,536	642,098	2,617,634	2.32	0.67	1.92
**2017**	414	62	476	2,046,047	678,559	2,724,606	2.02	0.91	1.75
**Total/average**	3603	437	4040	12,931,794	4,348,834	17,280,628	2.78	1.00	2.34

Table 2 shows the baseline characteristics of patients admitted for fall-related injuries. The mean age of patients remained the same (32.9 ± 18.26 years) across the study duration except for 2012 and 2015 (34 ± 18 years) and 2013 (30 ± 17 years). About three out of four fall-related injuries were among the 20–59 age group and 8% were aged 60 years and above. Patients under the age of 20 years represented 18% of the total fall-related injuries.

**Table 2:** Baseline characteristics of patients admitted for fall-related injuries between 2010 and 2017 (*N* = 4040)

Mean age ± SD	32.9 ± 18.26
**Age group in years** (*n* = 3994; 98.9%)^a^	
0–19	726 (18.2)
20–29	1043 (26.1)
30–39	949 (23.8)
40–49	640 (16.0)
50–59	310 (7.8)
60–69	153 (3.8)
70 and above	173 (4.3)
**Gender (*n*** **= 4040; 100%)**^a^	
Males	3603 (89.2)
Females	437 (10.8)
**Type of occupation (*n*** **= 2059; 50.9%)**^**a**^	
Laborer	1485 (72.1)
Installation, maintenance & repair	237 (11.5)
Transportation	79 (3.8)
Housekeeper	77 (3.7)
Agriculture	41 (2.0)
Military, protective services Management	23 (1.1)
Engineering	17 (0.8)
Management	12 (0.6)
Office & Administration	10 (0.5)
Other occupations^b^	78 (3.8)
**Location of fall (*n*** **= 3783; 93.6%)**^**a**^	
Work	1989 (52.6)
Home	1314 (34.8)
Street & public place	210 (5.6)
Recreational	138 (3.6)
Other	132 (3.4)
**Height of fall (meters) (*n*** **= 3764; 93.2%)**^**a**^	
Less than 1 m	1064 (28.3)
1.0–2.9 m	875 (23.2)
3.0–5.9 m	1152 (30.6)
≥ 6 m	673 (17.9)

Data expressed as numbers, and valid percentages in bracket

*SD* Standard deviation

^a^available data; ^b^other occupations include: sales, art, entertainment, sports, and food preparation and serving

More than half of the fall-related injuries (53%) occurred at workplace and 72% of them were laborers. However, nearly half (49%) of the data on the type of occupation was missing. Data on the height of fall was available in 93% of cases. Approximately 28% of the fall-related injuries were from the same level whereas 18% were from a height of 6 m or above.

Table 3 shows the clinical characteristics and outcomes of patients admitted for fall-related injuries Victims of fall-related injuries were otherwise healthy (82%); only 8% had hypertension, 7% diabetes mellitus, 3% congestive heart failure and very few had psychiatric illness (0.3%). Almost 4% of cases were alcohol consumers with blood alcohol concentration ranging from 1.1 to 130.2 mmol/L.

**Table 3:** Clinical characteristics and outcomes of patients admitted for fall-related injuries (*N* = 4040)

**Comorbidities** (*n* = 4040, 100%)^a^	
• Hypertension	320 (7.9)
• Diabetes mellitus	272 (6.7)
• Congestive heart failure	106 (2.6)
• Psychiatric illness	13 (0.3)
• None reported	3329 (82.3)
**Injured regions (*n*= 4040, 100%)**^a^	
• Head	1433 (35.5)
• Spine	1158 (28.7)
• Chest	946 (23.4)
• Pelvis	566 (14.0)
• Abdomen	526 (13.0)
**Injury characteristics**	
GCS at ED	15 (IQR 15–15)
Head AIS	3 (IQR 3–4)
Chest AIS	3 (IQR 2–3)
Abdomen AIS	2 (IQR 2–3)
Pelvis AIS	2 (IQR 2–2)
**ISS (*n*= 3954, 97.9%)**^a^	
• 1–8	1306 (33.0)
• 9–15	1657 (41.9)
• 16–24	578 (14.6)
• 25–49	397 (10.0)
• ≥50	16 (0.5)
TRISS	0.98 ± 0.05
RTS	7.5 ± 1.2
**Outcomes**	
Hospital LOS days (*n* = 4030, 99.8%)^a^	5 (IQR 2–11)
ICU stay days (*n* = 1018, 25.2%)	3 (IQR 2–8)
Ventilator days (*n* = 493, 12.2%)	4 (IQR 1–9)
In-hospital mortality	154 (3.1)

Data expressed as numbers and valid percentages in brackets, or mean ± standard deviation, or median with interquartile range (IQR) in bracket

*GCS* Glasgow coma score, *ED* Emergency Department, *AIS* Abbreviated injury scale, *TRISS* Trauma injury severity score, *RTS* Revised trauma score, *LOS* Length of stay, *ICU* Intensive care unit

^a^available data

Most of the injuries affected the head (36%) followed by spines (29%) and chest (23%). Head and chest injuries had median AIS score of 3 (range 1–6). Injuries to other body regions such as pelvis and abdomen were moderate in severity with median AIS score 2 (range 1–5). The ISS score revealed that most of the victims had moderate polytrauma (42%) followed by mild polytrauma in 33%. The median ISS was 9 (range 1–75). The mean RTS score was 7.5 ± 1.2 and the TRISS was 0.98 ± 0.05.

In-hospital outcome data showed that the median length of ICU, ventilator and hospital stays were 3 (1–126), 4 (1–43) and 5 (1–254) days respectively. Overall in-hospital mortality was 3.1%.

Table 4 shows the characteristics and outcomes of patients following fall-related injuries by gender between 2010 and 2017. Comparative analysis of fall-related injuries by gender demonstrated significant differences in the location of fall, height of fall, injured body regions and injury severity. Males were more frequently injured following falls in workplace whereas females were vulnerable at home. Falls resulted in injuries among males occurred more commonly from higher heights when compared to that in females. Males experienced more chest and spinal injuries in comparison to females. Head injuries were comparable among both groups; however, the GCS among males were significantly lower. Injury severity in terms of ISS was higher among males. There were no significant differences in outcomes in terms of the HLOS and mortality in both genders.

**Table 4:** Characteristics and outcomes of patients following fall-related injuries by gender between 2010 and 2017 (*N* = 4040)

	Males (*n* = 3603, 89.2%)	Females (*n* = 437, 10.8%)	*P*-value
Mean a**ge** ± **SD** (years)	33.2 ± 16.7	31.6 ± 27.9	0.26
**Location of fall**			0.001
**Work**	1935 (53.7)	54 (12.4)	
**Home**	1015 (28.2)	299 (68.4)	
**Street**/public place	185 (5.1)	25 (5.7)	
**Recreational**	111 (3.1)	27 (6.2)	
**Other**	357 (9.9)	32 (7.3)	
**Median height of fall** (meters)	3 (IQR 0.5–5)	0.5 (IQR 0–0.5)	0.001
**Head injury**	1284 (35.6)	149 (34.1)	0.53
**Chest injury**	881 (24.5)	65 (14.9)	0.001
**Spinal injury**	1069 (29.7)	89 (20.4)	0.001
**Abdominal**	490 (13.6)	36 (8.2)	0.001
**GCS at ED**	13.8 ± 3.3	14.3 ± 2.6	0.001
**ISS**	12.0 ± 8.7	9.8 ± 6.8	0.001
**TRISS**	0.98 ± 0.06	0.98 ± 0.03	0.002
**RTS**	7.5 ± 1.2	7.6 ± 0.9	0.03
**Hospital LOS** (days)	5 (IQR 2–11)	5 (IQR 2–12)	0.23
**Mortality**	123 (3.4)	8 (1.8)	0.08

Data expressed as numbers and valid percentages in bracket, or mean ± standard deviation (SD), or median with interquartile range (IQR) in bracket

*GCS* Glasgow coma score, *ED* Emergency Department, *ISS* Injury Severity Score, *TRISS* Trauma Injury Severity Score, *RTS* Revised Trauma score, *LOS* Length of stay

Table 5 shows the comparative analysis of fall-related injuries among 3 age groups. Patients in the working age group were the major victims of the fall-related injuries when compared to the other age groups and these falls were frequently from a height of approximately 3 m and above. The proportion of females in older adults who got injured following falls was significantly higher than other age groups. Older adults got injured more often following fall at home and these falls were frequently from the same level. Albeit, head injuries were significantly higher in the young age group. Chest injuries were more common among the older adults whereas spinal injuries and abdominal injuries were more often in the working age group. The mean GCS at ED was higher, and mean ISS was lower in the older adults. Worse outcomes including increased HLOS and mortality were observed more in the older adults.

**Table 5:** Characteristics and outcomes of patients following fall-related injuries by age-groups between 2010 and 2017 (*N* = 3994)

	0–19 yrs. (*n* = 726, 18.2%)	20–59 yrs. (*n* = 2942, 73.7%)	≥60 yrs. (*n* = 326, 8.1%)	*P*-value
Males	543 (74.8)	2794 (95.0)	225 (69.0)	0.001
Location of fall				
Work	39 (5.4)	1896 (64.4)	47 (11.3)	0.001
Home	469 (64.6)	601 (20.4)	237 (72.7)	
Street/public place	77 (10.6)	110 (3.7)	21 (6.4)	
Recreational	75 (10.3)	61 (2.1)	2 (0.6)	
Other	66 (9.1)	274 (9.3)	29 (8.9)	
Median height of fall (meters)	1 (IQR 0–2.5)	3(IQR 1.5–5.0)	0 (IQR 0–0.5)	0.001
Head injury	387 (53.3)	926 (31.5)	96 (29.4)	0.001
Chest injury	79 (10.9)	764 (26.0)	91 (27.9)	0.001
Spinal injury	65 (9.0)	1024 (34.8)	60 (18.4)	0.001
Abdominal	70 (9.6)	423 (14.4)	25 (7.7)	0.001
GCS at ED	14.3 ± 2.3	13.8 ± 3.3	14.3 ± 2.4	0.001
ISS	9.4 ± 7.8	12.3 ± 8.7	11.6 ± 7.1	0.001
TRISS	0.99 ± 0.04	0.98 ± 0.06	0.98 ± 0.06	0.001
RTS	7.6 ± 0.8	7.5 ± 1.1	7.6 ± 0.9	0.03
Hospital LOS (days)	2 (IQR 1–5)	6 (IQR 3–13)	9 (IQR 4–21)	0.001
Mortality	7 (1.0)	86 (2.9)	19 (5.8)	0.001

Data expressed as numbers and valid percentages in bracket, or mean ± standard deviation or median with interquartile range (IQR) in bracket

*GCS* Glasgow coma score, *ED* Emergency Department, *ISS* Injury severity score, *TRISS* Trauma injury severity score, *RTS* Revised trauma score, *LOS* Length of stay

Table 6 compares patients with age 20–45 years (74%) to patients of age above 45 years (26%). Young age was associated with higher incidence of fall, more work-related fall, more head injury and fewer mortality (2.6% vs 4.9%, *p* = 0.001) in comparison to the older age group.

**Table 6:** Characteristics and outcomes of patients with fall injuries among middle and old- age groups (*n* = 3268/4040)

	20–45 yrs. (*n* = 2430)	46–64 yrs. (*n* = 838)	*P*-value
Males	2313 (95.2)	706 (92.4)	0.001
Location of fall			
Work	624 (66.8)	309 (36.9)	0.001
Home	444 (18.3)	394 (47.0)	
Other	362 (14.9)	135 (16.1)	
Median height of fall (meters)	3 (IQR 2–5)	1 (IQR 0–3)	0.001
Head injury	787 (32.4)	235 (28.0)	0.02
Chest injury	591 (24.3)	264 (31.5)	0.001
Spinal injury	849 (34.9)	235 (28.0)	0.001
Abdominal	364 (15.0)	84 (10.0)	0.001
GCS at ED	13.7 ± 3.4	14.2 ± 2.7	0.001
ISS	12.4 ± 8.7	11.8 ± 8.2	0.09
TRISS	0.98 ± 0.06	0.97 ± 0.05	0.001
RTS	7.5 ± 1.2	7.6 ± 1.0	0.03
Hospital LOS (days)	6 (IQR 3–13)	6 (IQR 3–13)	0.59
Mortality	64 (2.6)	41 (4.9)	0.001

Data expressed as numbers, and valid percentages in bracket or mean ± standard deviation or median with interquartile range (IQR) in bracket

*GCS* Glasgow coma score, *ED* Emergency Department, *ISS* Injury severity score, *TRISS* Trauma injury severity score, *RTS* Revised trauma score, *LOS* Length of stay

In another subanalysis as shown in Supplementary table 1, patients in the age 20–34 years, 35–44 years and 45–64 years; had a mortality rate of 2.9%, 2.3, and 4.1% respectively (*p* = 0.11). However, longer HLOS was observed in both young age-groups (20–34 and 35–44 years) when compared to the age group of 45–64 years (*p* = 0.001).

The comparative analysis of fall-related injuries based on the height of fall is given in Table 7. The height of fall correlated with the severity of injury in terms of ISS and HLOS. Nearly 18% of the falls were from a height of 6 m or above with mortality rate of 6.4% whereas fall from height ≤ 1 m occurred at home and had a mortality 2.6%.

**Table 7:** Characteristics and outcomes of patients following fall-related injuries by height of fall categories between 2010 and 2017 (*N* = 3764)

	< 1 m (*n* = 1064, 28.3%)	1.0–2.9 m (*n* = 875, 23.2%)	3.0–5.9 m (*n* = 1152, 30.6%)	≥ 6 m (*n* = 673, 17.95)	*P*-value
Mean age ± SD (years)	37.2 ± 24.7	29.0 ± 17.3	32.8 ± 12.9	31.8 ± 11.4	0.001
**Age groups**					
**0–19 yrs.**	275 (25.8)	226 (25.8)	110 (9.5)	53 (7.8)	0.001
**20–59 yrs.**	566 (53.2)	621 (70.9)	1002 (86.9)	584 (86.8)	
**≥ 60 yrs.**	222 (20.9)	51 (5.8)	35 (3.0)	11 (1.6)	
Males	846 (79.5)	787 (89.9)	1106 (96.0)	632 (89.6)	0.001
Location of fall					
• Workplace	156 (14.7)	411 (47.0)	853 (74.0)	502 (74.6)	0.001
• Home	690 (64.8)	284 (32.5)	158 (13.7)	85 (12.6)	
• Other	218 (20.4)	180 (20.6)	141 (12.2)	86 (12.8)	
Head injury	436 (41.0)	337 (38.5)	344 (29.9)	208 (30.9)	0.001
Chest injury	171 (16.1)	181 (20.7)	303 (26.3)	246 (36.6)	0.001
Spinal injury	137 (12.9)	215 (24.6)	462 (40.1)	299 (44.4)	0.001
Abdominal	79 (7.4)	109 (12.5)	154 (13.4)	140 (20.8)	0.001
GCS at ED	14.4 ± 2.3	14.3 ± 2.5	13.9 ± 3.0	12.5 ± 4.6	0.001
ISS	10.2 ± 7.3	10.8 ± 7.7	12.3 ± 8.7	15.0 ± 10.6	0.001
TRISS	0.98 ± 0.04	0.99 ± 0.05	0.98 ± 0.06	0.97 ± 0.08	0.001
RTS	7.66 ± 0.74	7.64 ± 0.83	7.56 ± 0.98	7.01 ± 1.9	0.001
Hospital LOS (days)	4 (IQR 2–10)	4 (IQR 2–8)	6 (IQR 3–13)	8 (IQR 3–20)	0.001
Mortality	28 (2.6)	10 (1.1)	34 (3.0)	43 (6.4)	0.001

Data expressed as numbers and valid percentages in bracket, or mean ± standard deviation or median with interquartile range in bracket

*GCS* Glasgow coma score, *ED* Emergency Department, *ISS* Injury severity score, *TRISS* Trauma injury severity score, *RTS* Revised trauma score, *LOS* Length of stay

Table 8 illustrates the burden of fall-related injuries in Qatar based on previous studies. Although the objectives and study populations in these studies were different, fall was one of the main mechanisms of injuries.

**Table 8:** Burden of fall-related injuries in Qatar from previous studies

Study	Study population	Study duration	Total injuries	Fall injuries
El-Faramawy et al. (2012) [14]	Spinal fractures	2007 to 2009 (26 months)	442 spinal injuries	31% Fall from height
Tuma et al. (2013) [15]	WRFI	2007–2008 (12 months)	315 total falls	95% WRFI
El-Matbouly et al. (2013) [16]	Traumatic brain injury	2008–2011 (48 months)	1665 traumatic brain injury	35% Fall from height
Al-Thani et al. (2014) [2]	work-related injuries	2010–2012 (36 months)	1496 work-related injuries	51% Fall from height
Parchani et al. (2014) [17]	TSAH	2008–2012 (55 months)	403 TSAH	35% Fall from height
Mahmood et al. (2014) [3]	Intubated patients in TICU	2009–2010 (24 months)	343 Intubated in TICU	18% Falls
Alyafei et al. (2015) [4]	Pediatric trauma	2011 (12 months)	163	36%
Al-Thani et al. (2015) [18]	traumatic neck injury	2008–2012 (60 months)	51	10%
Arumugam et al. (2015) [19]	Abdominal trauma	2008–2011 (48 months)	1036	25%
El-Menyar et al. (2017) [20]	Pediatric traumatic brain injury	2010–2014 (60 months)	945 traumatic brain injury	22% Falls
Abdelrahman et al. (2018) [21]	fall at home	2008–2011 (36 months)	98 fall at home	2.2 of trauma admissions

Trauma registry was the data source in all these studies

*WRFI* Work-related fall injuries, *TASH* Traumatic subarachnoid hemorrhage, *TICU* Trauma intensive care unit

## Discussion

To the best of our knowledge, this is a unique study of its kind in the Arabian Gulf region that describes moderate to severe fall-related injuries based on a nationally representative data. The higher rate of fall-related injury was seen in patients at age between 20 and 34 years whereas the higher rate of fall-related mortality was at older age (45–64 years). Previously, multiple studies among trauma patients in Qatar revealed the burden of fall-related injuries, however, these were among some specific groups of patients including work-related trauma, pediatric trauma, head injuries, spinal injuries and neck injuries (Table 8) [2–4, 14–21]. The present study described the overall epidemiological and clinical characteristics of fall-related injuries in the country, and also based on gender, age groups and height of fall. It provided new data on the characteristics of fall-related injuries based on a nationally representative sample across all age groups which is of significance in providing important information for fall prevention programs.

The present study revealed that the rate of hospitalized fall-related injuries in Qatar was 2.34 per 10,000 population. The rate of fall injuries decreased by 39% from 2010 to 2017, however, the average number of patients per year still accounted for 32% of the moderate and severe injuries requiring admission. Working age-group of 20–59 years and males were the main victims, especially due to falls from greater height at workplace. Outcomes including longer HLOS and higher mortality were associated with the height of falls. Older adults were more frequently injured following falls at home from same level or less than 1 m height and had a higher mortality. Head injuries were more common among pediatric and young adults.

Hospital cost associated with traumatic injuries following falls in Qatar was published earlier which demonstrated that it costs approximately 15,735 USD per patient [15]. Our finding showed that t more than one out of three (32%) trauma admissions were fall-related, which could subsequently pose a significant impact on the health financial system in the country. It was demonstrated previously that 95% of the fall injuries in Qatar were work-related [15] and half of the total injuries at work-place were due to falls [2].

Tuma et al. study was on falls at the workplace among the construction workers and the large majority of the victims were migrant workers [15]. Notably, the majority of workforces in Qatar are migrants as expatriates represent around 85% of the country population [22]. It is evident from the literature that migrants are predominantly young males and their proportion in high risk occupations is higher especially among those who are coming from lower socioeconomic backgrounds [22]. Much of these low-income young male migrants in the Arabian Gulf countries work mainly as construction workers, drivers, custodians and numerous other positions [23, 24]. Recent reforms in the labor laws have been taken in the country to further strengthen the policies of safety practices that should be adopted in all work environments [25].

The present study, demonstrated that the rate of fall injuries decreased over the years which may be linked to the increased safety awareness among the construction workers and companies. In addition, the infrastructure projects that had started almost 10 years back in preparation for FIFA world cup football-2022 is currently at the final stage, and therefore exposure to work-related injuries reduced over the years.

Fall was the leading mechanism of injury among the pediatric trauma patients in Qatar which accounts for 36% of the total pediatric trauma [4]. El-Menyar et al. studied pediatric traumatic brain injuries (TBIs) and found that fall from height was the second leading mechanism of TBIs (22%) following motor vehicle crashes (MVCs) [20]. Both studies on pediatric trauma revealed that there was no significant difference in male preponderances across all pediatric age-groups [4, 20]. Although, the age-group classification was not specific to the pediatric population, our study demonstrated that pediatric group and younger adults (up to age of 19) accounted for 18% of the total falls in all age-groups.

Although the major victims of fall-related injuries in our study was among the younger age-group, older adults over 60 years (8%) could not be ignored because of the worse outcomes including increased length of hospital stay and mortality (5.8%) when compared to other age-groups. A previous study in Qatar explored falls at home among all age groups and found that older adults were more likely to experience worse consequences following bathroom falls [21]. Interestingly, female representation among this age group was higher when compared to patients under the age of 60 years [21]. Our study revealed that fall injuries at home were more frequent in females than males.

A recent study from China reported that slipping, tripping and stumbling resulting in falls on the same level was accounting for high mortality rate (29%) [26]. A study from Iran showed that the average HLOS among all age-group of fall victims were over 6 days and, in our study, the median HLOS was 5 days [27]. On the other hand, the median HLOS among the older adults was 9 days. Comorbidities of the older age group could contribute to the longer duration of hospital stay and mortality.

One of the major limitations of the present study is the retrospective design itself; however such retrospective study is much needed to provide an epidemiologic picture of an important public health problem to form better understanding and set better preventive measures. Secondly, the fall injuries in this study may include intentional falls along with the unintentional falls. This will not affect the main objectives of the study because of insignificantly low number of intentional falls (1.4%); however, it may have impact on the pattern of injuries. Thirdly, information on work-related falls are lacking, however data on location of falls including home, work-place, street and recreational were available. Therefore, injuries occurred at workplace was assumed as work-related. Recently, the trauma registry and other hospital records in our institute included the field of work-place injuries. Finally, more detailed classification of pediatric and very young adults could have been used to provide more accurate information on the burden of pediatric trauma; however, this is beyond the scope of the current paper.

## Conclusions

Although the rate of fall-related injuries in Qatar decreased over the years, it remains as a significant burden in trauma center since it accounts for 32% of the moderate to severe injuries requiring hospital admission. Epidemiologic and clinical characteristics, and in-hospital outcomes of the patients by gender, age-group and height of fall provides a knowledge base for effective preventive measures.

## Supplementary information

**Additional file 1: Supplementary table 1.** Subanalysis for fall-related injuries in patients aged 20–34 years, 35–44 years and 45–64 years.

## Data Availability

All data generated or analyzed during this study are included in this published article and its supplementary information files. Data are accessible upon agreement with the national trauma registry and the medical research centre at HMC.

## Abbreviations

ISS: Injury severity score
AIS: Abbreviated injury scale
GCS: Glasgow coma scale
FFH: Fall from height
TRISS: Trauma ISS
RTS: Revised trauma score
