# Supplementary material for: Epidemiological and clinical characteristics of fall-related injuries: a retrospective study
Source: BMC Public Health. 2020 Jul 29;20:1186. doi: 10.1186/s12889-020-09268-2 (PMC7388431; doi:10.1186/s12889-020-09268-2)
Supplement: Supplementary file 1 — Additional file 1: Supplementary table 1. Subanalysis for fall-related injuries in patients aged 20–34 years, 35–44 years and 45–64 years. [file 12889_2020_9268_MOESM1_ESM.docx]

| Supplementary table: Characteristics and outcomes of patients following fall-related in working age-groups (N=3041) | | | | |
| --- | --- | --- | --- | --- |
|  | **20-34 yrs.** (n=1555, 51.1%) | **35-44 yrs.** (n=799, 26.3%) | **45-64 yrs.** (n= 687, 22.6%) | **P-value** |
| Males | 1475 (94.9) | 765 (95.7) | 637 (92.7) | 0.03 |
| Location of fall  Work  Home  Other | 1063 (68.4)  264 (17.0)  228 (14.6) | 506 (63.3) 167 (20.9)  126 (15.8) | 355 (51.7)  219 (31.9)  113 (16.4) | 0.001 |
| Median height of fall (meters) | 3 IQR (2-5) | 3 IQR (1.5-5) | 2 (0-4) | 0.001 |
| Head injury | 510 (32.8) | 255 (31.9) | 188 (27.4) | 0.001 |
| Chest injury | 359 (23.1) | 207 (25.9) | 235 (34.2) | 0.001 |
| Spinal injury | 563 (36.2) | 262 (32.8) | 229 (33.3) | 0.20 |
| Abdominal | 250 (16.1) | 106 (13.3) | 82 (11.9) | 0.02 |
| GCS at ED | 13.7±3.5 | 13.9±3.1 | 13.8±2.9 | 0.01 |
| ISS | 12.6±8.9 | 11.9±8.0 | 12.1±8.7 | 0.12 |
| TRISS | 0.98±0.07 | 0.98±0.05 | 0.98±0.06 | 0.01 |
| RTS | 7.5±1.2 | 7.6±0.9 | 7.6±1.1 | 0.08 |
| Hospital LOS (days) | 6 IQR (3-13) | 6 IQR (3-14) | 5 IQR (3-12) | 0.001 |
| Mortality | 45 (2.9) | 18 (2.3) | 28 (4.1) | 0.11 |
| Data expressed as numbers, and valid percentages in bracket or mean± standard deviation or median with interquartile range (IQR) in bracket; GCS: Glasgow Coma Score; ED: Emergency Department; ISS:Injury Severity Score;TRISS: Trauma Injury Severity Score ; RTS: Revised Trauma score; LOS: Length of Stay | | | | |
